# Antifungal defensins and their role in plant defense

**DOI:** 10.3389/fmicb.2014.00116

**Published:** 2014-04-02

**Authors:** Ariane F. Lacerda, Érico A. R. Vasconcelos, Patrícia Barbosa Pelegrini, Maria F. Grossi de Sa

**Affiliations:** ^1^Department of Biochemistry and Molecular Biology, Federal University of Rio Grande do NorteNatal, Brazil; ^2^Plant-Pest Interaction Laboratory, Embrapa – Genetic Resources and BiotechnologyBrasília, Brazil; ^3^Catholic University of BrasiliaBrasília, Brazil

**Keywords:** plant defensins, antifungal, phytopathogens, peptide structure, peptide function, transgeny

## Abstract

Since the beginning of the 90s lots of cationic plant, cysteine-rich antimicrobial peptides (AMP) have been studied. However, Broekaert et al. (1995) only coined the term “plant defensin,” after comparison of a new class of plant antifungal peptides with known insect defensins. From there, many plant defensins have been reported and studies on this class of peptides encompass its activity toward microorganisms and molecular features of the mechanism of action against bacteria and fungi. Plant defensins also have been tested as biotechnological tools to improve crop production through fungi resistance generation in organisms genetically modified (OGM). Its low effective concentration towards fungi, ranging from 0.1 to 10 μM and its safety to mammals and birds makes them a better choice, in place of chemicals, to control fungi infection on crop fields. Herein, is a review of the history of plant defensins since their discovery at the beginning of 90s, following the advances on its structure conformation and mechanism of action towards microorganisms is reported. This review also points out some important topics, including: (i) the most studied plant defensins and their fungal targets; (ii) the molecular features of plant defensins and their relation with antifungal activity; (iii) the possibility of using plant defensin(s) genes to generate fungi resistant GM crops and biofungicides; and (iv) a brief discussion about the absence of products in the market containing plant antifungal defensins.

## INTRODUCTION

Plants are constantly exposed to several pests and pathogens in nature. They have developed complex defense mechanisms to protect themselves against the attack of pathogens (Gachomo et al., 2003, 2010). To circumvent these occurrences, defense factors are produced, including, hydrogen peroxide, phenolics, terpenoids, alkaloids, polyacetylenes, and a diverse array of pathogenesis-related (PR) defense proteins (Broekaert et al., 1997; Garcia-Olmedo et al., 1998; Osbourn, 1999; Van Loon et al., 2006; Benko-Iseppon et al., 2010) and plant defensins (Terras et al., 1995).

Defensins are small cationic peptides of 45–54 amino acid residues with a conserved signature of cysteines, which can form three to four disulfide bridges. Plant defensins exhibit a conserved tertiary structure that consists of a triple-stranded antiparallel β-sheet and one α-helix that are stabilized into a compact shape by the disulfide bridges. These bridges form a cysteine-stabilized α-helix β-sheet motif (CSα/β) (Kobayashi et al., 1991; Zhu et al., 2005). In addition to the CSα/β motif, two additional conserved motives, named α-core, encompassing the loop connecting the first β-strand to the α-helix, and the γ-core containing the hairpin loop that links β-strands 2 and 3 (Lβ2β3) were also present in the defensin structure (Yount and Yeaman, 2004; Yount et al., 2007). Despite the low level of amino acid sequence identity between defensins, their three dimensional structures are remarkably similar between different plant defensins (Pelegrini and Franco, 2005). Variations in the amino acids are reflected by small conformational changes in the tertiary structure that contribute to the broad range of biological activities in these proteins. Only one amino acid substitution can change the spectrum of activity exhibited by these peptides (Carvalho and Gomes, 2011).

Since the beginning of 1990s, lots of cationic plant cysteine-rich antimicrobial peptides (AMP) have been studied. Plant defensins were first described in the seeds of wheat (*Triticum turgidum*) and barley (*Hordeum vulgare*) (Colilla et al., 1990; Mendez et al., 1990). They were characterized as a new member of the thionine family due to their similarity in molecular mass, amino acid sequence and number of cysteines. However, subsequent studies performed by Bruix et al. (1995) revealed the existence of differences in the pattern of the disulfide bridges, demonstrating that these two peptide families are unrelated. Broekaert et al. (1995) renamed these peptides as “plant defensins,” after comparing their structural and functional resemblance to previously characterized AMPs found in insects and mammals.

## DEFENSINS AND THEIR CONTRIBUTION TO PLANT DEFENSE

### HOW CAN DEFENSINS HAVE A ROLE IN PLANT DEFENSE?

The role of defensins in the preformed defense of plants is well reported. Several reports show that defensins are an integral part of the plant innate immune system. Most plant defensins already characterized show a constitutive pattern of expression with up regulation in response to pathogen attack, injury and some abiotic stresses (de Beer and Vivier, 2011).

Several features make clear that defensin peptides are involved in plant defense (Selitrennikoff, 2001). Their distribution is consistent with their putative defense role. They have been identified in leaves, tubers, flowers, pods and seeds, playing an important role in the protection of germinating seeds and developing seedlings (Garcia-Olmedo et al., 1998). In addition, plant defensins are also localized in the xylem, stomata, and stomata cells, parenchyma cells, and other peripheral areas (Kragh et al., 1995; Segura et al., 1998; Chen et al., 2002). The presence in the different tissues is consistent with a defensive role of such peptides, once it is believed that such sites are the place of the first contact with a potential pathogen (Carvalho and Gomes, 2011).

Moreover, plant defensins have a broad spectrum of *in vitro* antimicrobial activity and, currently, there are several reports describing the production of transgenic plants constitutively expressing foreigner defensins. Hence, they possess an enormous multiplicity of biological activities, such as antimicrobial, insecticidal, inhibiting protein synthesis, mediating abiotic stress, and Zn tolerance, and as inhibitors of digestive enzymes (Carvalho and Gomes, 2009, 2011). According to Franco (2011), these defense peptides are classified as promiscuous proteins, as they show numerous biological activities. As an example, there is the family of defensins isolated from *Vigna unguiculata*, in which different homologous forms may act as antifungal, antibacterial, and enzyme inhibitors (Franco, 2011). Although they present multiple functions, the antimicrobial activity of plant defensins is mainly observed against fungi.

Therefore, the present review explores the current knowledge about the structure and mechanism of action of plant defensins with emphasis on its activity against phytopathogenic fungi. Furthermore, we describe the current use of these peptides as biotechnological tools in the production of transgenic plants that could result in the future release of agronomically important crops resistant to various diseases.

## STRUCTURAL CONFORMATION AND MECHANISM OF ACTION

Plant defensins present a well-conserved three-dimensional structure composed by a cysteine-stabilized α/β (CSαβ) motif, which forms one α-helix followed by three anti-parallel β-sheets. The amino acid sequence is also quite conserved, especially due to the presence of six to eight cysteine residues, which form three to four disulfide bridges in the sequence of Cys1-Cys8, Cys2-Cys5, Cys3-Cys6, and Cys4-Cys7 (Lay and Anderson, 2005). Nevertheless, plant defensins with five disulfide bonds have been described, such as the peptide from *Petunia hybrida* (PhD1), whose cysteine residues interact in the following order: Cys1-Cys10, Cys2-Cys5, Cys3-Cys7, Cys4-Cys8, and Cys6-Cys9 (Janssen et al., 2003). The additional disulfide bond does not affect the typical three-dimensional structure of the defensin, which is located after the α-helix and the first β-sheet (Janssen et al., 2003).

Furthermore, plant defensins with alternative structures have been identified in the literature, including defensins from *Nicotiana alata* (NaD1), *Petunia hybrida* (PhD1 and PhD2), and ZmESR6 isolated from developing maize kernels. These defensins contain an extra acidic C-terminal prodomain whose function is still unknown, although it has been suggested that it is involved in vacuolar targeting or in eliminating potential detrimental effects caused by the basic nature of the defensin (De Coninck et al., 2013).

**FIGURE 1:**
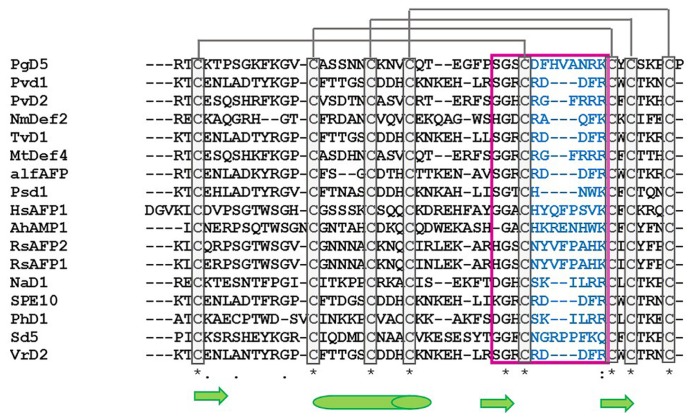
**Alignment of the amino acid sequence of antifungal plant defensins.** PgD5: *Picea glauca* defensin (Accession: AAR84643); Pvd1: *Phaseolus vulgaris* defensin 1 (Accession: ADR30066); PvD2: *Phaseolus vulgaris* defensin 2 (Accession: ADR3006); NmDef1: *Nicotiana megalosiphon* defensin (Accession: ACR46857); TvD1: *Tephrosia villosa* defensin (Accession: AAX86993); MtDef4: *Medicago trunculata* defensin 4 (Accession: 2LR3_A); alfAFP: *Medicago sativa* antifungal peptide 1 (Accession: AAG40321); Psd1: *Pisum sativum* defensin 1 (Accession: 1JKZ_A); HsAFP1: *Heuchera sanguinea* antifungal peptide (Accession: P0C8Y5); AhAMP1: *Aesculus hippocastanum* antimicrobial peptide 1 (Accession: AAB34970); RsAFP1: *Raphanus sativus* antifungal peptide 1 (Accession: 1AYJ_A); RsAFP2: *Raphanus sativus* antifungal peptide 2 (Accession: P30230); NaD1: *Nicotiana alata* defensin 1 (Accession: 4ABO_A); SPE10: *Pachyrrihizus erosus* peptide (Accession: 3PSM_A); PhD1: *Petunia hybrida* defensin 1 (Accession: 1N4N_A); Sd5: *Saccharum officinarum* defensin 5 (Accession: 2KSK_A); VrD2: *Vigna radiata* defensin 2 (Accession: 2GL1_A). Asterisk indicates conserved cysteine amino acid residues among antifungal defensins (gray boxes). Gray lines represents the disulfide bridges between cysteine amino acid residues. Pink box and blue amino acid residues correspond to the γ-core region. Green arrows indicate β-sheet region and green cylinder indicate α-helix region. Alignment was done using ClustalW2 Tool.

**FIGURE 2:**
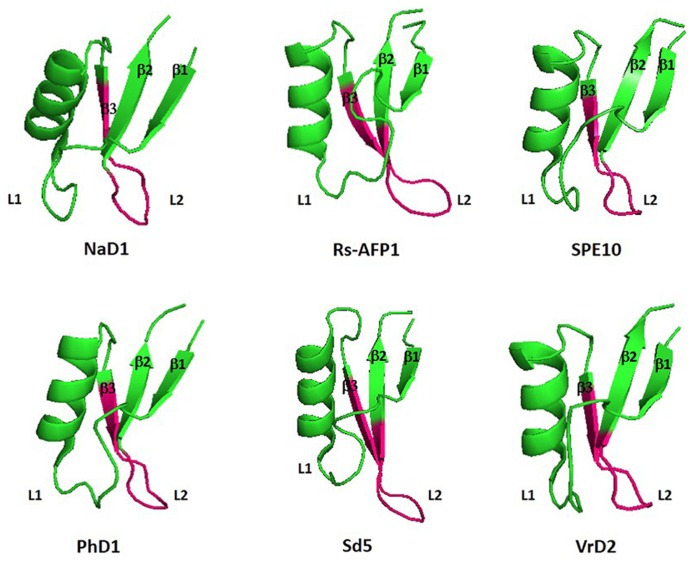
**Three-dimensional structure of six antifungal defensins from plants.** Pink region highlight the γ-core motif of each peptide. β1: β-sheet 1; β2: β-sheet 2; β3: β-sheet3; L1: Loop1; L2; Loop 2: NaD1: *Nicotiana alata* defensin 1 (Accession: 4ABO_A); Rs-AFP1: *Raphanus sativus* antifungal peptide 1 (Accession: 1AYJ_A); SPE10: *Pachyrrihizus erosu* peptide (Accession: 3PSM_A); PhD1: *Petunia hybrida* defensin 1 (Accession: 1N4N_A); Sd5: *Saccharum officinarum* defensin 5 (Accession: 2KSK_A); VrD2: *Vigna radiata* defensin 2 (Accession: 2GL1_A). All figures were designed using PyMol Molecular Graphic System Version 1.2r3pre, Schrödinger, LLC.

As they are peptides consisting of 45–54 amino acid residues, structural studies on crystallography and nuclear magnetic resonance (NMR) have been widely extended during the last few years. Among the peptides with antifungal activity, whose structures have been elucidated, are included the defensins from *Nicotiana alata* (NaD1), *Pachyrrhizus erosus* (SPE10), *Petunia hybrida* (PhD1), *Pisum sativum* (Psd1), *Raphanus sativus* (Rs-AFP1), and *Saccharum officinarum* (Sd5) (Fant et al., 1998; Almeida et al., 2002; Janssen et al., 2003; Lay et al., 2003; de Paula et al., 2011; Song et al., 2011; Van der Weerden and Anderson, 2013; **Figure 1**). An amino acid sequence alignment of antifungal defensins from plants shows that they do not present conservative amino acid sequences, except the cysteine residues and a glycine residue positioned in the second β-sheet (Pelegrini and Franco, 2005; Van der Weerden and Anderson, 2013). According to their structural features, plant defensins show a conserved γ-core signature classified as the dextromeric isoform, which is related to the amino acid sequence conservation of the region NH_2_…[X_1-3_]-[GXC] = [X_3-9_]-[C]…COOH (**Figures 1** and **2**). This preservation in the primary sequence gives them a three-dimensional conformation denominated γ-core motif, consisting of two antiparallel β-sheets, with an interpolated turn region. Earlier studies classified plant defensins as belonging to the β-γ-α Group, according to their relative structural γ-core (Yount and Yeaman, 2004). It has been described that the γ-core motif is important for antimicrobial activity in disulfide-stabilized peptides (Yount and Yeaman, 2004), not only for their cysteine content, but especially due to the presence of positively charged residues at the second β-turn of their structure (Fant et al., 1998). This characteristic was first observed when the structure of *R. sativus* defensin 1 (Rs-AFP1) was determined by ^1^HNMR, and mutation analyzes was also performed using the peptide isoform Rs-AFP2 (De Samblanx et al., 1997; Fant et al., 1998). In both cases, it was demonstrated that positively-charged amino acids located at the γ-core motif were essential for the antifungal activity of theses peptides, and the substitution of neutral residues inside this γ-core by other positively-charged amino acid residues increased their activity towards pathogenic fungi. Spelbrink et al. (2004), while studying defensins from *Medicago trunculata*, verified that the antifungal activity of MtDef1 was due to the presence of four positively-charged amino acids, also located in the γ-core region, which was lacking in the structure of the non-antifungal peptide MtDef2. Moreover, *in vitro* assays revealed that this region might be involved in the ability of MtDef1 to block L-type Ca++ channels in mammalian cells.

There are two major hypothesis that tries to explain the mechanism of action of antimicrobial defensins: (i) the carpet model and (ii) the pore model. In both models, defensins are described to interact with the negatively charged molecules present at the cell membrane of pathogens, causing an increase of its permeabilization, leading to cell leakage and death by necrosis. While the carpet model emphasizes the pore formation of several peptides into the membrane, the pore model shows that peptides form oligomers that, then, form multiple pores into the cell membrane. However, there is an alternative hypothesis, where defensins do not damage the cell membrane, but interact with the phospholipids, leading to an increase of ion permeability, or even to the transportation of these peptides to the intracellular environment (Wilmes et al., 2011; Hegedus and Marx, 2013). Hence, they can also enhance reactive oxygen species (ROS) and activate programmed cell death (PCD; Wilmes et al., 2011; Hegedus and Marx, 2013).

Moreover, positively charged amino acid residues were described to be important for antifungal activity, when located at loops and β-sheet regions. Hence, it was observed that the concave side of the VI β-turn from Rs-AFP1 was positively-charged, leading to the suggestion that the contact of this peptide with pathogenic fungi may occur through electrostatic interactions (De Samblanx et al., 1997; Fant et al., 1998). Other studies on the structural analyses of plant defensins, such as NaD1, described the importance of positively-charged amino acid residues at the loop region between β2 and β3, not only for antifungal activity, but for also functioning as a specificity factor towards different pathogens (Lay et al., 2003). Recently, it was reported that the amino acid residues located in the γ-core motif of MtDef4 are key tools for its antifungal activity and its specificity towards pathogenic fungi (Sagaram et al., 2011). First, *in vitro* assays using only the γ-core sequence of Mtdef4 and MsDef1 (alfAFP) showed that the high content of positively charged residues with the core of MtDef4 could, alone, provide antifungal activity, in contrary to the core of alfAFP, which was inactive against filamentous fungi (Sagaram et al., 2011). Later, mutagenesis studies on the region RGFRRR from MtDef4 showed that the substitution of the hydrophobic and positively-charged residues, Phe and Arg, at positions 3 and 4, respectively, by Ala residues decreased intensely its activity against fungi. Furthermore, it was shown that both defensins present differences on their kinetics of permeabilization, when assayed against *Fusarium graminearum*, as MtDef4 was able to induce a more potent antifungal activity and could take up the molecular probe SYTOX Green (SG) at a dependent concentration, indicating physical damage of cell membranes. In comparison, alfAFP induced a less effective membrane permeabilization, and did not induce a concentration dependent SG uptake (Sagaram et al., 2011).

**FIGURE 3:**
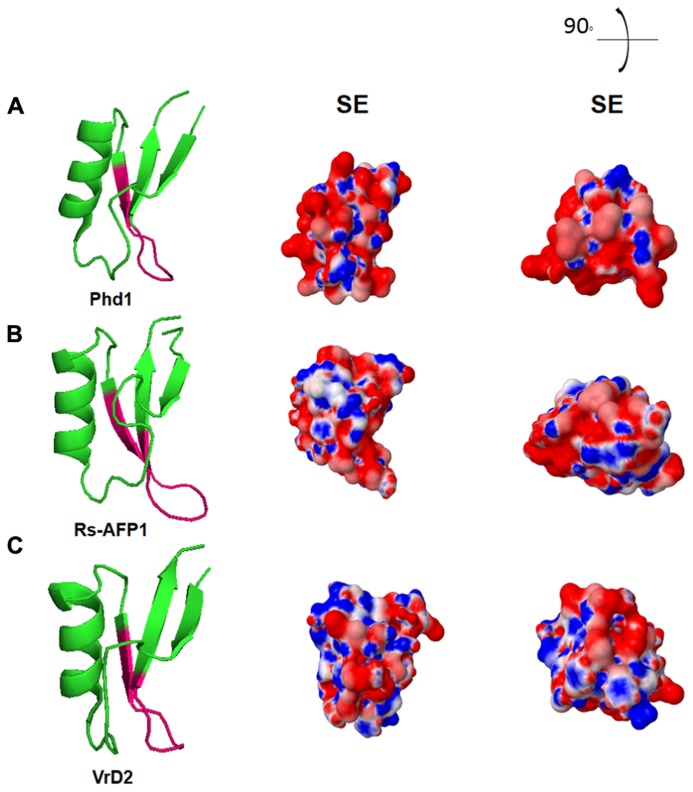
**Electrostatic surface of three plant defensins under vacuum environment.** Three-dimensional structures of **(A)** Phd1; **(B)** Rs-AFP1; **(C)** VrD2. SE: solvent excluded electrostatic surface. 90° and the illustration and the right top of the figure indicates the angle deviation for new visualization of the peptides structures. All figures were designed using PyMol Molecular Graphic System Version 1.2r3pre, Schrödinger, LLC.

Further reports displayed a comparison between the electrostatic potential surfaces of different defensins with their potential antimicrobial activities (Almeida et al., 2002). However, although there was no pattern of charge distribution among defensins, there was a high indication that plant defensins may act as potassium channel inhibitors, due to their similarities with neurotoxins, which contains residues for such activity (Almeida et al., 2002). **Figure 3** shows the electrostatic surface area of three antifungal plant defensins (Phd1, Rs-AFP1, and VrD2), in which the site related to the second loop of the defensins that contains the γ-core region is described as the most important site for their antifungal activity. This is highly positively-charged in the cartoons where electrostatic surfaces were designed in vacuum. Therefore, it corroborates with the antifungal assays and the *in silico* studies performed by many researchers over the last 20 years.

A structural study on sugarcane defensin, Sd5, provided new information about the mechanism of action for those antifungal peptides. It was described that the hydrophobic core at the C-terminal of the defensin is also important for membrane interaction and permeabilization (de Paula et al., 2011). In addition, evaluations on the backbone conformational dynamics of Sd5 suggest that the mechanisms of its structural exchange is related to modifications in the hydrogen bond distances of the β-sheet and α-helix of the peptide, giving it the ability to bind to membranes. Hence, membrane permeabilization and vesicle leakage induced by Sd5 may occur through the interaction of the side chains of residues of three serines and the glycosyl part of the membrane model with glucosylceramide extracted from the hyphae of *F. solani* (de Paula et al., 2011). Recent studies on dynamics of Sd5 structure revealed that this peptide displays many dynamic properties. It was able to interact with a sphingolipid glycosylceramide (CMH) membrane in a conformational selection process, which involved a specific binding, while other flexible regions of Sd5 showed to interact with the interface in a nonspecific manner (Valente et al., 2013).

**FIGURE 4:**
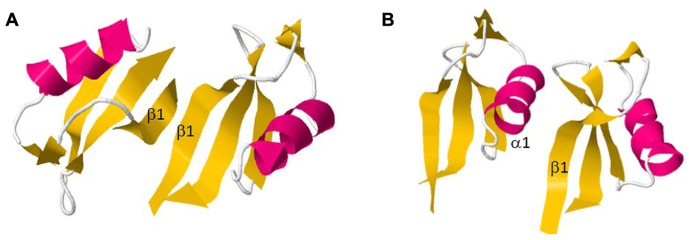
**Dimer formation of two plant defensins.** **(A)** NaD1; **(B)** SPE10. All figures were designed using PyMol Molecular Graphic System Version 1.2r3pre, Schrödinger, LLC.

Recent reports described the structural conformation of dimeric defensins being highly significant for its antifungal activity (Song et al., 2011; **Figure 4**). In this way, analyses of the defensin from *Pachyrrihizus erosus*, SPE10, provided the selection of the binding pattern Arg36-Trp42-Arg40 as essential for dimer formation. Moreover, it was demonstrated that Trp42 is fundamental for antifungal activity of plant defensins, as it is absent in non-antifungal peptides Therefore, dimers of SPE10 are arranged in a side-by-side manner with the α-helix of one monomer interacting with the β-sheet of the second monomer, leading to a stretched and twisted molecular surface. Conformational changes on Arg36 and Trp42 would alter the dimeric interface of SPE10, destabilizing the dimer (Song et al., 2011). In addition, the dimerization of the defensin NaD1 was performed in order to evaluate the relation between structural conformation and antifungal activity. In contrary to what was observed by SPE10 dimer, monomers of NaD1 were connected by a β-sheet/β-sheet configuration, although the antifungal activity was maintained (Lay et al., 2012; **Figure 4**). Hence, plant defensins that form dimers coupled with their positively charged surface area become highly efficient molecules against pathogenic fungi, as they can strongly interact with the negatively charged glycoproteins located at the fungal cell walls (Lay et al., 2012).

## TARGETED FUNGI AND EFFECTIVE CONCENTRATIONS

One of the first studies that attempted to highlight this class of plant antimicrobial defensins was carried out with two peptides isolated from Radish seeds, Rs-AFP1 and Rs-AFP2. Both peptides were assayed against 20 different plant pathogenic fungi and the lower protein concentration required for 50% inhibition of fungal growth (IC_50_) was obtained by Rs-AFP2, when assayed against *Pyricularia oryzae.* Its IC_50_ ranged from 0.08 to 5 μM. Since that lots of defensins were reporter to show high biological activity in the range of micromolar to nanomolar as will soon be shown. Terras et al. (1992) were the first ones to report the importance of disulfide bonds to defensins stabilization and the role of inorganic ions in its antifungal activity. They also showed how thermostable defensins are, once they found that heating Rs-AFP1 and 2 at 100°C for 10 min did not affect antifungal properties of such molecules. The stability of such molecules is an important feature which allows wondering a wide range of biotechnological applications to plant defensins.

Few years after Terras report, Osborn et al. (1995) increased the knowledge about plant defensins and their effects under fungi. They assayed four AMP isolated from *Aesculus hippocastanum* (Ah-AMP1)*, Clitoria ternatea* (Ct-AMP1), *Dahlia merckii* (Dm-AMP1), *Heuchera sanguinea* (Hs-AFP1) against eight different fungi in the presence, or absence, of inorganic ions. The lower IC_50_, around 0.1 μM, was acquired when Ah-AMP1 was tested towards *Cladosporium sphaerospermum*, *Leptosphaeria maculans*, and *Septoria tritici*. Hs-AFP1 presents the same antifungal activity when assayed against *Septoria tritici*. In all the studies, inorganic ions decreased IC_50_. When visualized under a microscope, it was possible to see that such antifungal peptides caused distinct morphological changes during germ tube elongation and hyphae development, like multiple hyphae buds or the diminished of the rate of germ tube elongation (Osborn et al., 1995; **Table 1**).

**Table 1:** Short sample of plant defensins and its IC_**50**_ concentration against its fungal targets.

Plant defensin	Organism	Target organism	IC_50_(μM)	Reference
PgD5	*Picea glauca*	*Verticillium dahliae*	0,4	Picart et al. (2012)
Defensin-like peptide	*Phaseolus vulgaris*	*Mycosphaerella arachidicola*	3,9	Wong et al. (2012)
NmDef02	*Nicotiana megalosiphon*	*Fusarium oxysporum*	1	Portieles et al. (2010)
Pdc1	*Zea mays*	*Fusarium graminearum*	0,75	Kant et al. (2009)
Limyin	*Phaseolus limensis*	*Fusarium solani*	8,6	Wang et al. (2009)
TvD1	*Tephrosia villosa*	*Pheaoisariopsis personata*	1,9	Vijayan et al. (2008)
MtDef4	*Medicago truncatula*	*Fusarium graminearum*	0,75	Ramamoorthy et al. (2007)
MsDef1(alfAFP)	*Medicago sativa*	*Fusarium graminearum*	1,2	Spelbrink et al. (2004)
Psd1	*Pisum sativum*	*Neurospora crassa*	2	Almeida et al. (2001)
alfAFP	*Medicago sativa*	*Verticillium dahliae*	1	Gao et al. (2000)
HsAFP1	*Heuchera sanguinea*	*Septoria tritici*	0,1	Osborn et al. (1995)
AhAMP1	*Aesculus hippocastanum*	*Leptosphaeria maculans*	0,1	Osborn et al. (1995)
RsAFP2	*Raphanus sativus*	*Pyricularia oryzae*	0,08	Terras et al. (1992)

A great number of the earlier studies about the mechanism of action of plant defensins agree on the membrane permeabilization outcome (Thevissen et al., 1996, 1999). More recently, two peptides similar to plant defensins were reported to show such disruption power. The first one, from *Phaseolus vulgaris*, permeabilizes *Mycosphaerella arachidicola* membrane, among other fungi (Wong et al., 2012). The second, from *Picea glauca*, was reported to act on permeabilization of *Verticillium dahlia* membranes (Picart et al., 2012). Membrane permeabilization seems to be just one of a huge variety of mechanism of action for such molecules. While some results point to cellular membranes as the point of action, others suggest intracellular targets (Thevissen et al., 2000).

The use of antifungal peptide genes to generate important agronomical traits resistant to fungal disease have been seen with some skepticism by the biotechnological thinkers. Plant defensins proved to be useful for biotechnological purposes in the year of 2000, when Gao et al. (2000) showed that AlfAFP, an antifungal peptide from *Medica sativa* and active towards *Verticillium dahliae*, was expressed in a transgenic potato, increasing resistance against such filamentous fungus. The IC_50_ of AlfAFP towards *Verticillium dahlia* was determined at 1 μM, around ten times higher than the previous AMP described here (Gao et al., 2000). However, the resistance of transgenic potato expressing AlfAFP towards *Verticillium dahliae* showed to be more effective in greenhouse conditions and in the field than the chemical methods, what make of it a useful choice to plant transformation aiming resistance to phytopathogenic fungi, which will be discussed in detail later in this review.

Almeida et al. (2001) reported the heterologous expression of a *Pisum sativum* defensin (Psd1) in a eukaryotic expression, system based on the methilotrophic yeast *Pichia pastoris*. The high amount of Psd1 produced by *P. pastoris* expression system (13.8 mg/L), allowed investigations about the conformational features between wide type and recombinant form of Psd1 (rPsd1). Besides being active towards filamentous fungi, such as *Neurospora crassa*, Psd1 did not demonstrate any activity against yeasts, even at high (20 μM) concentrations (Almeida et al., 2000). According to the report, the heterologous expression in *Pichia pastoris* did not significantly affect the defensin conformational features, and all post-translational modifications needed to its activity had been done. One of the small differences between Psd1 and rPsd1 was their N-terminal sequences. rPsd1 kept four amino acids residues from the recombinant signal peptide, and this seemed to be related to the 5-fold decrease on its activity towards *F. solani* and *Aspergillus niger*, in comparison to the wide type peptide. rPsd1 activity towards *N. crassa* was not affected, which suggests distinct modes of action of Psd1 against fungi belonging to different classes (Almeida et al., 2001). Furthermore, the *Pichia pastoris* system was also used to produce the recombinant *Nicotiana megalosiphon* defensin (NmDef02) active against *F. oxysporum* (Portieles et al., 2010).

Plant defensins have also been expressed in prokaryotic system and tested against fungi. TvD1, a defensin from *Tephrosia villosa*, was expressed in *Escherichia coli* and assayed towards *Pheaoisariopsis personata* (Vijayan et al., 2008; **Table 1**). A comparison between the expression of Pdc1, a corn defensin, in yeast and *E. coli* was done and in both cases the peptide kept its antimicrobial activity, however, Pdc1 expressed in yeast (IC_50_ 7.5 μM) was more efficient than when expressed in *E. coli* (IC_50_ 30 μM) in arrest *F. graminearum* growth. The presence or absence of a His-tag also influences its activity, suggesting that defensins are sensible to covalent modifications on its terminal ends (Kant et al., 2009).

Different from some results, which suggest the importance of N-terminus in defensin activity (Almeida et al., 2001), Spelbrink et al. (2004) demonstrated that the major determinant of antifungal activity of a defensin from *Medicago sativa* (MsDef1) resides in the carboxy-terminal region. They evaluated six different defensin chimeras obtained from molecular combinations of MsDef1, active towards *F. graminearum*. They also analyzed MtDef2, a defensin from *Medicago truncatula*, which did not have any activity towards *F. graminearum*. Among the six chimeras, only the ones harboring the MsDef1 portion on the C-terminal displayed some activity against *F. graminearum* (Spelbrink et al., 2004). The divergence among results pointing to C-terminus and to N-terminus as essentials to plant defensin activity, expose the uncertainty about the relation between structure and function of such molecules and even more on its modulation mechanism of activity.

Three years after Spelbrink findings, Ramamoorthy et al. (2007) tried to go a little deeper into the cellular mechanisms of activity modulation using Medicago defensins against *F. graminearum*. They have demonstrated that mutants of *F. graminearum* can react differently to Medicago defensins MsDef1 and MtDef4. *F. graminearum* mutant, whose MAP Kinase cascades were disrupted, were hypersensitive to MsDef1. However, it did not show any difference on its sensitivity to MtDef4. MAP kinase signaling cascades seemed to provide protection towards MsDef1, but not to MtDef4, which suggests that these plant defensins utilize specific signaling pathways to alter fungal growth (Ramamoorthy et al., 2007).

Besides antimicrobial activity, a plant defensin from *Phaseolus limensis* named Limyin and active against *F. solani*, were also reported to show antiproliferative activity towards human tumor cells (Wang et al., 2009), suggesting there are lots of things to be discovered about the cellular targets and mechanisms of action of plant defensins.

Plant defensins encompass a class of biomolecules with the potential to be explored as biotechnological tools towards phytopathogenic fungi, which nowadays, are controlled only by chemicals. The wide natural sources of these molecules and the heterogeneity of their action on different targets allow hundreds of possible biotechnological approaches that, together with their low effective concentration, as shown in **Table 1**, could lead to phytopathogeninc fungi control with less environmental impact.

## BIOTECHNOLOGICAL APPLICATIONS AND TRANSGENY

Although there are many transformed plants in the market with additional genes coding to proteins that confer resistance towards herbicides and insect-pests, there is still no transgenic plant available against phytopathogenic fungi, nor even containing plant defensins as the resistant factor. Nevertheless, several studies describe the efficient activity of antifungal defensins when transformed into different host plants (**Table 2**). Therefore, plant defensins with antifungal activity have become the first molecule for the development of transgenic crops resistant to phytopathogens.

**Table 2:** Antifungal defensins from plant sources used for transformation into foreign species.

Peptide	Origin of peptide	Transformed plant	Pathogenic fungi tested	References
Rs-AFP2	Radish	Tobacco	*Alternaria longipes*	Terras et al. (1995)
		Apple	*Fusarium culmorum*	De Bondt et al. (1999)
		Tomato	*Alternaria solani*	Parashina et al. (2000)
			*Fusarium oxysporum*	
			*Phytophtora infestans*	
			*Rhizoctonia solani*	
		Pear		Lebedev et al. (2002)
		Rice	*Magnaporthe oryzae*	Jha and Chattoo (2010)
			*Rhizoctonia solani*	
Pea defensin	Pea	Canola	*Leptosphaeria maculans*	Wang et al. (1999)
D4E1	Synthetic	Tobacco	*Aspergillus flavus*	Cary et al. (2000)
			*Verticilium dalhia*	
BSD1	Stamen	Tobacco	*P. parasitica*	Park et al. (2002)
BjD	Mustard	Tobacco	*F. moniliforme*	Anuradha et al. (2008)
			*P. parasitica*	
		Peanut plants	*Cercospora arachidicola*
			*Pheaoisariopsis personata*	
Wasabi defensin	Wasabi	Rice	*Magnaporthe grisea*	Kanzaki et al. (2002)
alfAFP	Alfalfa	Potato	*V. dalhiae*	Gao et al. (2000)
		Tomato	*R. solanacearum*	Chen et al. (2006)
MsDef1	Medicago sativa	Tomato	*F. oxysporum*	Abdallah et al. (2010)

The first attempt to evaluate of transgenic plants containing foreigner antifungal defensin genes was done in tobacco plants expressing Rs-AFP2, a peptide from radish. High levels of peptide expression were observed in the transformed tobacco plants, as well as an increasing resistance towards the phytopathogenic fungus *Alternatia longipes* (Terras et al., 1995). Four years later, the same defensin was used for studies with transgenic apple plants and evaluation against pathogenic fungi species (De Bondt et al., 1999). Hence, after transformation through *Agrobacterium tumefaciens*, the transgenic plants were selected and the expressed peptide was isolated and quantified. *In vitro* assays showed that the recombinant peptide was able to inhibit the germination of *Fusarium culmorum* spores (De Bondt et al., 1999). Tomato lines have also been transformed with Rs-AFP2, generating the increase in their antifungal activity. In this study, leaves of tomato plants overexpressing the radish defensin were extracted and tested against some phytopathogenic fungi, including *Alternatia solani*, *F. oxysporum*, *Phytophthora infestans*, and *Rhizoctonia solani* (Parashina et al., 2000). It was demonstrated that the crude extract of tomato leaves containing the radish defensin could decrease the activity of all the fungi cited above.

Furthermore, in 2002, Rs-AFP2 was again evaluated in transgenic plants, this time using two pear cultivars – Burakovka and Pamyat’ Yakoyleva. After transformation, leaves of pear plants were collected for PCR and Western Blot Hybridization analyses. The presence of the foreigner gene and recombinant peptide were detected through the respective techniques, confirming the success of plant transformation (Lebedev et al., 2002). Nevertheless, *in vitro* and *in vivo* assays against pathogen fungi are still to be done in order to check the antifungal activity of transgenic pear plants expressing Rs-AFP2. The most recent work on Rs-AFP2 was published in 2010, when Jha and Chattoo (2010) transformed this peptide into rice (*Oryza sativa* L. cv. *Pusa* basmati 1). The transgenic plants were tested *in vitro* and *in vitro* against *Magnaporthe oryzae* and *Rhizoctonia solani*, the main causes of rice losses in agriculture, revealing that overexpression of Rs-AFP2 can control the rice blast and sheath blight diseases (Jha and Chattoo, 2010).

In addition, other works on transgenic plants expressing an antifungal defensin were published. Hence, it was demonstrated that pea defensins transformed into *Brassica napus* cultivars enhanced their resistance against *Leptosphaeria maculans*, which causes blackleg diseases in plants (Wang et al., 1999). Tobacco plants transformed via *Agrobacterium tumefasciens* and containing a synthetic antifungal gene was also performed. The expressed peptide, named D4E1, provided an increasing resistance of tobacco against *Aspergillus flavus* and *Verticillium dahlia* (Cary et al., 2000). Tobacco was also used for transformation of the stamen defensin BSD1, where the expressed peptide provided higher tolerance to the plant against the attack of *Phytophtora parasitica* (Park et al., 2002). Transformation of tobacco with the mustard defensin – BjD – once more validated the potential of these peptide-family members as excellent antifungal agents, as transgenic plants displayed improved resistance towards *F. moniliforme* and *Phytophtora parasitica* (Anuradha et al., 2008). More recently, a defensin purified from maize, ZmDEF1, when transformed into tobacco plants, showed increased tolerance against *Phytophtora parasitica* (Wang et al., 2011). Transgenic peanut plants, expressing the same mustard defensin, also provided an enhancement of tolerance against *Cercospora arachidicola* and *Pheaoisariopsis personata*, which mutually cause the late leaf spot disease (Anuradha et al., 2008).

Kanzaki et al. (2002) performed a successful attempt of expressing a defensin from *Wasabia japonica* into rice plants, as an effort to increase the plant resistance against the phytopathogenic fungus *Magnaporthe grisea*. Moreover, they showed that T3-generation transformed rice plants could still overexpress the wasabi defensin and maintain its ability to control *Magnaporthe grisea in vivo*. Earlier, it was demonstrated that transgenic potato expressing an antifungal defensin from alfalfa (alfAFP) was more resistant to the attack of *Verticillium dahliae*, when compared to non-transformed plants (Gao et al., 2000). A summary of information of expressed plant defensins into plant cultivars can be seen at **Table 2**.

An attempt at transforming two different genes at the same time in tomato plants was performed using genetic material of a defensin and a glucanase from alfalfa, in order to analyze their efficiency towards phytopathogenic fungi. Therefore, T1-generation transgenic plants revealed enhanced tolerance to *R. solanacearum*, when compared to non-transformed plants, indicating the existence of a synergic effect of both proteins as antifungal molecules in tomato cultivars (Chen et al., 2006). Further efforts using other plant defensins into transformed tomato plants were carried out. In this way, Abdallah et al. (2010) inserted the *Medicago sativa* defensin gene into *Licopersicum esculentum* cultivar CastleRock and evaluated the transformed plants against the pathogenic fungus *F. oxysporum* f. sp. *Lycopersici. In vivo* assays demonstrated that T1- and T2-generations of transgenic tomato plants presented increased resistance against the fungal pathogen, when compared to non-transformed plants.

Plant defensins have also displayed indirect responses towards phytopathogenic fungi in transgenic plants, when other foreigner genes are being overexpressed (Murad et al., 2007). Hence, earlier reports showed that a peptide from *Arabidopsis thaliana*, named AtPep1 stimulated the transcription activation of the defensin gene *pdf1.2* (Huffaker et al., 2006). When AtPep1 precursor gene PROPEP1 was expressed into transgenic *Arabidopsis* plants, the transcription of PDF1.2 was also observed. Moreover, the expressed defensin stimulated root development, which, consequently, improved plant resistance against the filamentous fungus *Pythium irregular* (Huffaker et al., 2006).

Similar results were obtained when an ionotropic glutamate receptor (RsGluR) was transformed into *Arabidopsis* plants. The expression of RsGluR led to an up-regulation of defensins, causing an increase of the plant resistance towards *Botrytis cinerea* (Kang et al., 2006). Microarray analyses later confirmed that up-regulated defensins and jasmonic acid-responsive genes were produced after overexpression of RsGluR in *Arabidopsis*. Furthermore, the same plant species was transformed with a cotton non-symbiotic hemoglobin for tolerance against fungal pathogens. However, the foreigner gene could also induce a constitutive expression of the PR protein K (PR-1) as well as the defensin PDF1.2, providing an enhanced resistance to *Verticillium dahliae* (Qu et al., 2006).

## CONCLUSION AND PERSPECTIVES

Plant defensins correspond to a world of possibilities for defense mechanisms, and new peptides with different activities are still to be discovered, as well, studies with thousands of plant species need to be performed. Nowadays, several peptides already show satisfactory efficacy against such pathogens with strong potential to be applied for the production of a commercial fungicide or application into transgenic plants. But, the question remains. Why is there still no product containing antifungal plant defensins – in its natural form or in nanocapsules – already available in the market?

It is interesting that plant defensins with antifungal peptides are mostly studied for pathogens located in tropical areas, including Latin American, African, and some Asian countries. Moreover, the loss of commercially important crops due to the attack of phytopathogenic fungi is considered worldwide, until now, less detrimental than the losses caused by drought stress and insect-pests. Therefore, the efforts focused on the release of novel plant varieties resistant to drought stress and insect-pests are more significant, as there is mounting pressure to control these adversities in order to provide an increase in crop production. However, the development of transgenic plants expressing antifungal defensins or the production of defensin-based biofungicide depends, mainly, on the determination of regional research teams focusing on specific fungal targets, so these products can reach the market.

Furthermore, there is a long process required for analyzing the efficiency, environmental risks, safety towards animal and human consumption, and reproducibility of transformed plants expressing certain molecules, as well as the need for having an extremely stable, effective, and easy-to-produce peptide to be used in the fabrication of a biofungicide. Therefore, it is possible that there are already plant defensin-based products on the horizon that will soon be released on to the market.

Also, it is expected that, in the near future, antifungal defensin-based commercial agro-products be targeted as essential for the increase of crop production. This will stimulate and accelerate the transition between biotechnological research and field application of bioproducts.

## Conflict of Interest Statement

The authors declare that the research was conducted in the absence of any commercial or financial relationships that could be construed as a potential conflict of interest.
